# Brain-infiltrated monocyte macrophages in a rat model of temporal lobe epilepsy: revisiting the pro-inflammatory paradigm

**DOI:** 10.3389/fimmu.2025.1695856

**Published:** 2025-12-09

**Authors:** Wanda Grabon, Nadia Gasmi, Anatole Lang, Anne Ruiz, Béatrice Georges, Victor Blot, Michaël Ogier, Sylvain Rheims, Fabrice P Navarro, Laurent Bezin

**Affiliations:** 1 Université Claude Bernard Lyon 1, CNRS UMR5292, Inserm U1028, Centre de Recherche en Neurosciences de Lyon, TIGER Team, Bron, France; 2 Epilepsy Institute IDEE, 59 boulevard Pinel, Bron, France; 3 Université Claude Bernard Lyon 1, CNRS UMR5292, Inserm U1028, Centre de Recherche en Neurosciences de Lyon, GenCyTi, Bron, France; 4 French Armed Forces Biomedical Research Institute (IRBA), Brétigny-sur-Orge, France; 5 Department of Functional Neurology and Epileptology, Hospices Civils de Lyon and Lyon 1 University, Lyon, France

**Keywords:** temporal lobe epilepsy, microglia, infiltrating monocytes, neuroinflammation, brain cell sorting

## Abstract

Neuroinflammation is central to temporal lobe epilepsy, yet the specific role of myeloid cells remains unclear. In status epilepticus (SE) models, circulating monocytes infiltrate the brain, although distinguishing them from microglia is challenging. Using a rat model, we traced infiltrating monocytes post-SE to investigate their persistence, phenotypic evolution during epileptogenesis and contribution to neuroinflammation. By tracking phagocytosed fluorescent nanoparticles and performing CD68 immunohistochemistry, we confirmed that monocytes entered the brain in significant numbers 24 hours post-SE, after the inflammatory peak occurred (7 hours). Their long-term presence and evolution into monocyte-macrophages (mo-mΦs) up to 7 weeks were monitored histologically using CD68. Distinct inflammatory profiles were further characterized after cell enrichment and fluorescence-activated cell sorting (FACS) distinguishing monocytes/mo-mΦs (CD11b^+^CD45^hi^CD11a^hi^) from microglia (CD11b^+^CD45^lo^CD11a^lo^). Microglia were the main drivers of the early pro-inflammatory response, with TNFα transcript levels nearly 14-fold higher in microglia than in monocytes 24h post-SE. In contrast, 24h post-SE, infiltrating monocytes transiently displayed an anti-inflammatory and neuroprotective phenotype, being the main source of IL-10, showing ~4-fold higher CD206, and expressing Arg1 absent in microglia. Tracked up to 7 weeks, these cells progressively adopted a microglia-like phenotype, contributed to the microglial scar and, although their expression of pro-inflammatory markers resembled nonactivated microglia, we hypothesize that their persistent presence might fuel the low-grade inflammation typical of chronic epilepsy. Importantly, since infiltrating monocytes initially engage in a transient anti-inflammatory response, strategies aiming to sustain or enhance this protective role in the context of epileptogenesis and epilepsy may open promising avenues for therapeutic intervention.

## 1 Introduction

Neuroinflammation plays a critical role in the pathophysiology of temporal lobe epilepsy (TLE), influencing epileptogenic network formation post-injury, cognitive impairments, and the maintenance of spontaneous seizures (1–4). Both experimental models and TLE patients show evidence of blood–brain barrier (BBB) leakage (5–7) and monocyte infiltration, particularly within the hippocampus (8–11). Initial studies of these infiltrating monocytes suggested that they play a major role in the early extensive secretion of pro-inflammatory cytokines after pro-epileptogenic brain aggression, such as *status epilepticus* (SE), and that they may play a detrimental role in the development of TLE (10–12). However, the fate of these infiltrating monocytes and their role in sustaining inflammation remain debated (8, 10). Through noninvasive tracking studies—crucial both for minimizing the animal suffering caused by SE induction procedures and for maintaining a physiological approach—and for controlling postmortem tissue inflammatory responses (13), we provide new insights into the role of myeloid cells in post-SE inflammation and in established epilepsy.

Detecting infiltrating monocytes in the brain presents challenges due to their shared embryonic origin with microglia, resulting in nearly identical phenotypes (14, 15), and their potential evolution into brain monocyte-macrophages (mo-mΦs) (16, 17). To accurately track monocyte entry and fate, monocyte markers must be both specific to monocytes and sustained over time. Markers such as CCR2, CX3CR1, and Ly6C have allowed differentiation between microglia and infiltrating monocytes at early post-SE stages in mice (8, 10–12, 18). However, the role of infiltrating monocytes has received much less attention in rat models of SE, predominantly due to the lack of reliable markers. Moreover, while monocyte infiltration has been documented for weeks or even months in models of multiple sclerosis (19), spinal cord injury (19), and Alzheimer’s disease (20), the long-term fate of monocytes post-SE remains unexplored.

This study aimed to elucidate the fate of peripheral monocytes infiltrating the brain following SE induced by pilocarpine in young adult rats. By using circulating monocyte depletion strategies, fluorescent latex bead labeling, and fluorescence-activated cell sorting (FACS), we tracked the fate and persistence of monocytes during the chronic epilepsy phase up to 7 weeks after SE. Additionally, we used immunodetection of CD68, a marker specific to mo-mΦs in rats (21, 22) and humans (23) at the histological level, to monitor their presence and phenotypic changes. A secondary objective was to distinguish the respective contributions of microglia, infiltrating monocytes and mo-mΦs to the inflammatory response from the onset of epileptogenesis to the chronic phase of epilepsy. We assessed the inflammatory profiles of microglia, monocytes, mo-mΦs, and other brain cells and quantified inflammatory and neuroprotective marker transcripts via RT–qPCR while minimizing *ex vivo* transcription and translation during the dissociation and sorting process (13, 24).

## 2 Materials and methods

The full details of the methods are given in the **Supplementary Material**.

### 2.1 Experimental design

Four distinct groups of rats were similarly subjected to pilocarpine-induced SE at P42. The experimental design is illustrated in **Supplementary Figure S1**.

Group 1 – Tracking of infiltrating monocytes following clodronate-induced depletion. Nine rats were used to track fluorescent-labeled monocyte infiltration following monocyte/macrophage depletion induced by clodronate administration. The brains were collected at 1, 3 and 6 days following SE for subsequent immunohistology studies (SE + 1D, n=3; SE + 3D, n=3; SE + 6D, n=3).

Group 2 – Detection of brain microglia, infiltrating monocytes and differentiated monocyte–macrophages during epileptogenesis and chronic epilepsy. In thirty-one rats, myeloid, microglial and monocyte markers were detected via fluorescence immunohistochemistry during epileptogenesis; 7 h, 1 day, 6 days and 9 days following SE; and 7 weeks following SE in the hippocampus. CTRL, n=5; SE + 7 h, n=4; SE + 1D, n=5; SE + 6D, n=4; SE + 9D, n=4; CTRL + 7 W, n=4; SE + 7 W, n=5.

Group 3 – Tissue inflammation at the molecular level during epileptogenesis and chronic epilepsy, as determined via RT–qPCR. Thirty-nine rats were used to evaluate inflammatory profiles at the transcript level during epileptogenesis 7 h, 1 day and 9 days following SE and during chronic epilepsy, i.e., 7 weeks following SE in the hippocampus (CTRL, n=6; SE + 7 h, n=6; SE + 1D, n=6; SE + 9D, n=7; CTRL + 7 W, n=6; SE + 7 W, n=8).

Group 4 – Contribution of microglia and monocytes to neuroinflammation during epileptogenesis and chronic epilepsy via flow cytometry. Fourteen rats were used to evaluate the inflammatory status of the sorted microglia and monocyte/monocyte-macrophage mixture via flow cytometry. The brains were collected during epileptogenesis 1 day and 9 days following SE and during chronic epilepsy, i.e., 7 weeks following SE for subsequent tissue dissociation and cell sorting (CTRL, n=3; SE + 1D, n=3; SE + 9D, n=3; CTRL + 7 W, n=2; SE + 7 W, n=3). Once the cells were sorted, the inflammatory profiles of the cell populations were evaluated via RT–qPCR.

### 2.2 Animals

All animal procedures were in compliance with the guidelines of the European Union (directive 2010–63), followed French law (decree 2013/118) regulating animal experimentation, and were approved by the ethical committee of the Claude Bernard Lyon 1 University (protocol # BH-2008–11). Male Sprague–Dawley rats (Harlan, France, and Envigo, France) were housed in a temperature-controlled room (23 ± 1 °C) under diurnal lighting conditions (lights on from 6 a.m. to 6 p.m.) with water and food *ad libitum*.

### 2.3 Pilocarpine-induced status epilepticus

SE was induced by pilocarpine injection at P42. To prevent peripheral cholinergic side effects, scopolamine methylnitrate (1 mg/kg in saline, s.c.) was administered 30 min before pilocarpine hydrochloride (350 mg/kg, in saline, i.p.) was administered (25). After 2 h of continuous behavioral SE, diazepam (10 mg/kg, i.p.) was injected, followed 60 min later by a second injection of diazepam (5 mg/kg) to terminate the behavioral seizures. Given the severity of the model, the animals received special care as described in the **Supplementary Material**. Furthermore, this recognized high severity, which can lead to significant pain and distress, strongly influenced our decision to employ less invasive cell tracking methods (e.g., nanoparticle labeling and immunohistochemistry) over more aggressive alternatives (e.g., bone marrow transplantation), in strict accordance with ethical guidelines to minimize additional animal suffering and maintain the physiological relevance of our experimental setup.

### 2.4 Clodronate-induced monocyte/macrophage depletion and fluorescent nanoparticle labeling

Animals received clodronate liposomes (5mg/mL, i.p.) at the dosage of 1 mL per 100g body weight (26, 27). Clodronate was administered as a single dose 3 days before SE. Then, rats underwent SE and received 6 hours after its onset an injection of fluoresbrite YG carboxylate microspheres (0.5 μm diameter) via the tail vein (9.1 x 10^10^particles per rat).

### 2.5 Immunohistology

Brain slices – Forty-micron-thick coronal sections were cut from frozen paraformaldehyde (PFA)-fixed brains via a cryomicrotome.

Fluorescent immunolabelling – Detection and morphology appreciation of myeloid cells was performed with mouse anti-Cluster of differentiation 11b (CD11b) antibody (1:2000, CBL1512Z, Millipore). Detection of microglia was performed with goat anti-ionized calcium binding adaptor molecule 1 (Iba1) antibody (1:500, ab5076, Abcam). Infiltrating monocytes and monocyte-macrophages (mo-MΦ) were labelled with mouse anti-CD68 antibody (1:1000, MCA341GA, Bio-Rad). The fluorescent secondary antibodies used are listed in the **Supplementary Material**. Nuclei were stained with DAPI (300 nM, Molecular Probes).

Microscopy – Whole sections were scanned with a Carl Zeiss Axio Scan. Z1 Digital Slide Scanner (ZEISS) with an X20 lens on a 6 µm stack, using the pilot Zen (ZEISS), or observed with a TCS SP5X confocal microscopy system (Leica). Images were processed with Fiji software (ImageJ). The counting of round vs. branched cells was performed automatically via the ‘Analyze Particle’ function of Fiji software (size: 20-infinity pixels, circularity: 0.8:1.00). Double-positive Iba1/CD68 cells were quantified using the same “Analyze Particles” function, following colocalization detection with the “Image Calculator” (“AND”) operation. The percentage of double-positive cells among CD68-positive cells was then calculated.

### 2.6 Reverse transcription and real-time quantitative PCR

Tissues from perfused brains were crushed via a TissueLyser II (Qiagen) according to the manufacturer’s instructions. Total RNA from brain structures was extracted via Tri-Reagent LS. Total mRNAs were then reverse transcribed to complementary DNA (cDNA) via both oligo dT and random primers with the PrimeScript RT Reagent Kit (Takara, #RR037A) according to the manufacturer’s instructions in the presence of synthetic external nonhomologous poly(A) standard messenger RNA (SmRNA) to normalize the RT step, as previously described (28). Each cDNA of interest was amplified via a Rotor-Gene Q thermocycler (Qiagen), a SYBR Green PCR kit (Qiagen, #208052) and oligonucleotide primers (Eurogentec) specific to the targeted cDNA (**Supplementary Table S1**). The cDNA copy number detected was determined via a calibration curve, and the results are expressed as the cDNA copy number/µg total RNA.

The pro)inflammatory index (PI-I) and anti-inflammatory index (AI-I) were calculated via a specific set of pro-inflammatory and anti-inflammatory genes, namely, IL-1β, IL-6, and TNFα, and IL-4, IL-10, and IL-13, respectively, via the formula given in the **Supplementary Material** (13).

### 2.7 Flow cytometry

The samples were processed for tissue dissociation and cell sorting immediately following brain collection as quickly as possible on ice. To prevent any artifactual *ex vivo* gene expression changes during brain dissociation and cell sorting procedures, all buffers and solutions used during the process (from animal perfusion to sorted cell flash freezing) were supplemented with an “inhibition cocktail” composed of actinomycin D (3 µM), anisomycin (100 µM) and triptolide (10 µM), i.e., transcription and translation inhibitors (13, 24).

Brain tissue dissociation – Once collected, the tissues were cut into smaller pieces with a scalpel and processed for dissociation via Miltenyi Adult Brain Dissociation Kit (#130–107–677) according to the manufacturer’s instructions, running program 37C_ABDK_01. The inhibition cocktail was added to each reagent. Following debris removal, the cells were counted manually (with trypan blue) before magnetic sorting.

CD11b-positive cell magnetic enrichment – To increase fluorescence-activated cell sorting (FACS) yields and efficiency, cell suspensions were first enriched via the magnetic-activated cell sorting (MACS) technique, in which CD11b-positive cells (microglia and infiltrating monocytes) were magnetically separated for subsequent FACS via CD11b/c microbeads according to the manufacturer’s instructions (Miltenyi #130-105-634) from other cells before direct freezing. Details are provided in the **Supplementary Material**.

FACS – Microglia (CD11b^+^CD45^lo^CD11a^lo^) and infiltrating monocytes (CD11b^+^CD45^hi^CD11a^hi^) were sorted with a BD FACS Aria™ III Cell Sorter (BD Biosciences), as detailed in the **Supplementary Material**. The gating strategy is presented in **Supplementary Figure S2**.

### 2.8 Statistical analysis

Statistical analyses were performed via Prism 10.0 software (GraphPad, USA), as detailed in the **Supplementary Material**. The results are presented as the mean ± SEM (standard error of the mean). Differences with a p value<0.05 (p<0.05) were considered statistically significant. Details of the statistical tests for each figure are presented in the **Supplementary Data** (**Supplementary Table S2**).

## 3 Results

### 3.1 Myeloid cell reactivity in the hippocampus following pilocarpine-induced SE

In addition to the rapid microglial response observed following SE, pioneering studies have shown that monocytes infiltrate the brain parenchyma. However, the long-term fate of these infiltrating cells has not yet been established. CD11b immunodetection enables a detailed assessment of myeloid cell morphology, reflecting their state of activation and indicating their phenotype, as illustrated in the whole hippocampus from 7 h to 7 weeks post-SE (**Supplementary Figure S3**). In slices from healthy control rats, resting CD11b-positive microglia displayed a ramified morphology. Rapidly (7 h) after SE, almost all the microglia were in a reactive state, with an enlarged cell body and retracted ramifications. One day after SE, monocyte-like round CD11b cells, namely, those in the hilus of the dentate gyrus (DG), could be distinguished from reactive bushy cells (arrowheads, **Supplementary Figure S3F**). At this stage, no CD11b-positive cells recovered a resting morphology in the hippocampus. Nine days post-SE, a CD11b+ hypersignal, which we refer to as a microglial-like scar, was clearly visible in the hilus of the DG and CA1 (**Supplementary Figures S3G, H**). However, this signal persisted predominantly in the CA1 region at 7 weeks post-SE during the chronic phase of epilepsy (**Supplementary Figures S3I, J**), whereas CD11b-positive microglia returned to their basal morphological state in other areas of the hippocampus.

### 3.2 Peripheral origin of round-shaped CD11b-positive cells

**Figure 1:**
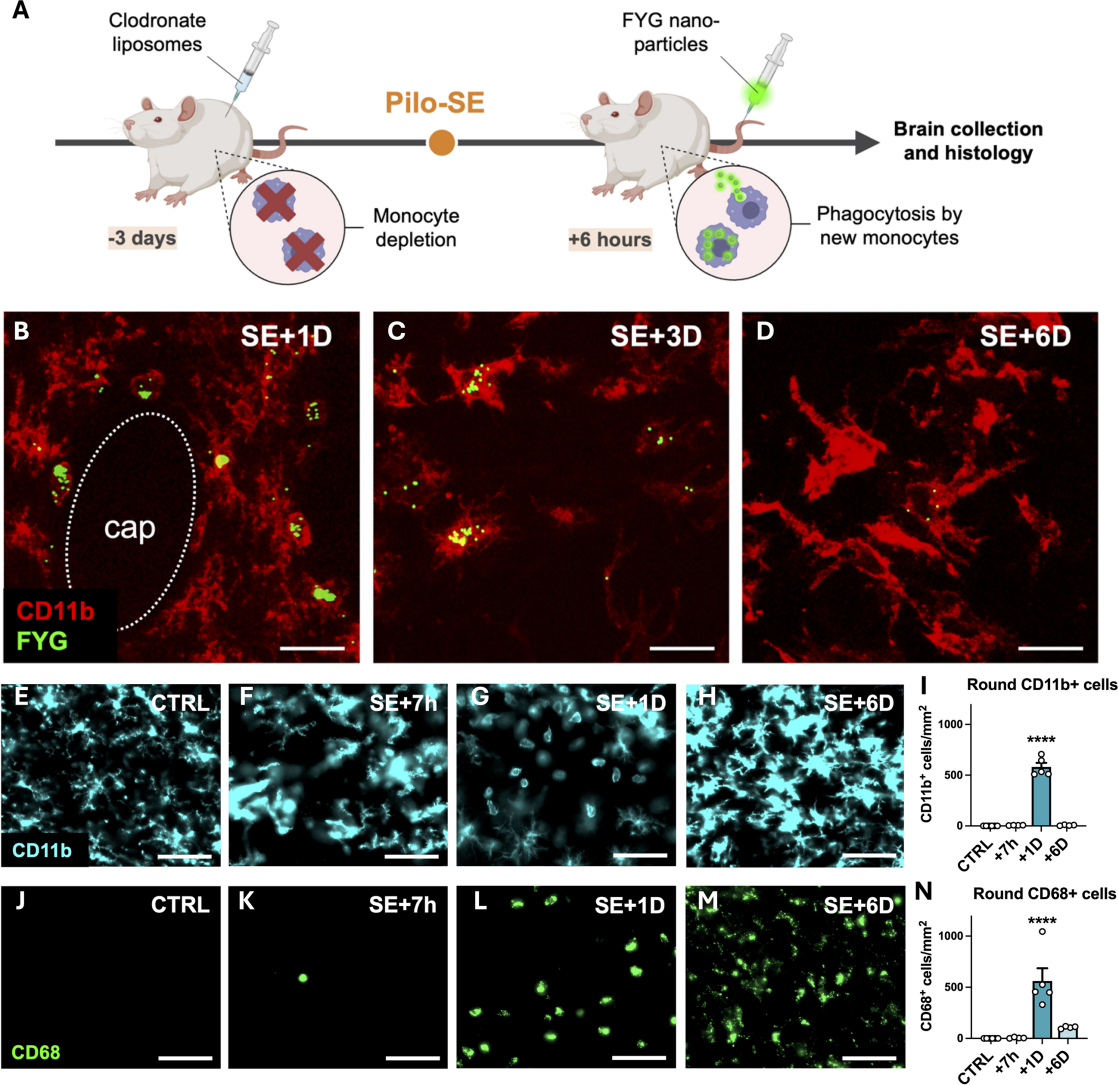
Peripheral monocytes infiltrate the hippocampus following SE between +7 h and +6 days and differentiate into brain monocyte-macrophages. **(A-D)** Fluorescent YG carboxylate microspheres (FYG, 0.5 µm) were injected into the tail vein 6 h after SE. No labeled monocytes could be detected in the brain parenchyma unless circulating monocytes were depleted with clodronate liposomes (1 ml/100g; i.p.) administered prior to SE. The rats were sacrificed 1D, 3D and 6D after SE. Detection of CD11b (red, CBL1512Z, Millipore) and FYG (green) in infiltrating monocytes at I day [**(B)** cap—capillary], brain monocyte-macrophages with extending processes at 3 days post-SE **(C)** and in cells resembling activated microglia at 6 days post-SE **(D)** in the hilus. Scale: 20 µm. **(E–N)** CD11b (E— l, cyan, CBL1512Z, Millipore) and CD68 [**(J–N)** green, MCA341GA, Bio-Rad] were immunodetected in the dentate gyrus following SE (CTRL, n=6; SE+7 h, n=4; SE+ID, n=5; SE+6D, n=4). Scale: 50 µm. Round CD11b-positive cells **(J)** and CD68-positive cells **(N)** were quantified in the dentate gyrus. The data were analyzed with Tukey's test following one-way ANOVA. The data are presented as the means ± SEMs. vs. CTRL. p<0.0001.

The presence of CD11b-positive round cells 24 h post-SE coincides with the early transient induction of the chemokines monocyte chemoattractant protein 1 (MCP-1) and macrophage inflammatory protein α (MIP1α) in the hippocampus (**Supplementary Figure S4**). These findings suggest that many of these round cells are likely infiltrating monocytes, although we cannot entirely rule out the possibility that some might be amoeboid microglia. To demonstrate the peripheral origin of round CD11b-positive cells and track them as long as possible in the brain tissue after SE, we tagged peripheral monocytes prior to the induction of SE. We first depleted circulating monocytes/macrophages by administering clodronate liposomes (29) 3 days before the induction of SE. We then intravenously administered Fluoresbrite yellow green (FYG) nanoparticles to label newly generated circulating monocytes (**Figure 1A**). We used the CD11b marker to detect myeloid cells, as at the onset of this study, no specific marker was available in rats to reliably distinguish microglia from infiltrating monocytes *in situ*. Detection of CD11b and FYG was performed at 1-, 3- and 6-days post-SE in the hilus and provided evidence that infiltrating monocytes remained in the hippocampus for at least 6 days post-SE. We observed that at 1-day post-SE, CD11b-positive round-shaped cells located in the blood vessels adjacent to the vascular wall were also positive for FYG (**Figure 1B**). At 3 days post-SE, fluorescent nanoparticles were found in CD11b-positive cells whose morphology was similar to that of activated microglia (**Figure 1C**). At 6 days post-SE, FYG nanoparticles were still observed in CD11b-positive cells (**Figure 1D**) but at a lower density, which may be explained by the fact that the CD11b-positive cells that had phagocytosed the FYGs may have processed or degraded them. Notably, fluorescent nanoparticles were not detected in Iba1-positive cells (data not shown). Therefore, at 6 days, the CD11b-positive cells labeled with FYG were likely cells resulting from the evolution of monocytes into brain mo-mΦs. The condition of the rats treated with clodronate prior to the induction of SE deteriorated beyond that of the rats subjected to SE alone, reaching the predefined endpoints 6 days post-SE, which prevented continuation of the study beyond this time.

The CD68 marker has recently emerged histologically as a specific marker of monocytes in rats, particularly in epileptogenic situations (21), which we confirmed here via Iba1 and CD68 costaining (**Supplementary Figure S5**). Unfortunately, the time elapsed between conducting the experiment on clodronate-FYG rats and identifying CD68 as a monocyte marker was too long, leading to complete degradation of FYG fluorescence and rendering CD68-FYG codetection impossible. Nevertheless, when numerous round CD11b-positive cells were detected 24 h post-SE in the hippocampus, numerous round CD68-positive cells were also detected in adjacent sections, with similar numbers (**Figures 1E–N**). No or few CD68-positive cells were detected in control brain sections or 7 h post-SE, respectively (**Figures 1J, K**). The number of CD11b-positive round cells quantified in the dentate gyrus between 7 h and 6 days post-SE was strongly associated with the number of CD68-positive round cells (simple linear regression, p<0.0001, R^2^ = 0.8043, y = 0.9255x + 40.11).

### 3.3 Some monocyte–macrophages persist in the hippocampus in the long term once epilepsy has developed

**Figure 2:**
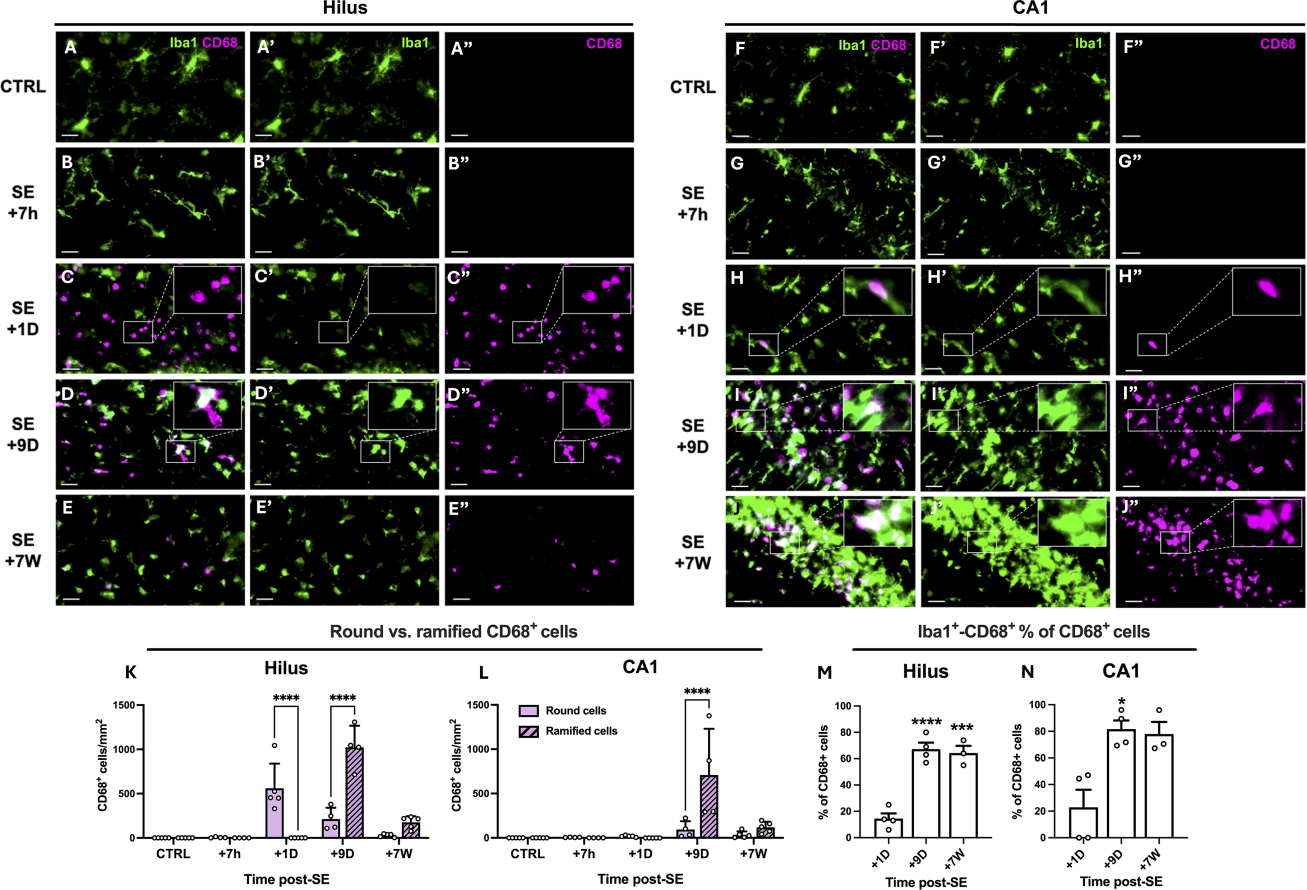
Infiltrating monocytes differentiate into brain monocyte-macrophages bearing morphological feature of microglia. **(A–J)**. CD68 (MCA341GA, Bio-Rad) and Iba1 (ab5076, abcam) were immunodetected in the dentate gyrus (left) and CA1 region (right), in control rats and 7 h, 1D, 9D and 7 W post-SE. Images were acquired with a slide scanner, objective ×20. Scale bars: 20 µm. **(K, L)**. Quantification of round and ramified CD68-positive cells in the hilus and in CA1 region, CTRL, n=5; SE+7 h, n=4; SE+1D, n=5; SE+9D, n=4; SE+7 W, n=5. Data are analyzed with Tukey’s test following two-way RM ANOVA. **(M, N)**. Percentage of Iba1-positive cells among CD68-positive cells was quantified in the hilus (M) and in CA1 region (N), CTRL, n=3; SE+7 h, n=3; SE+1D, n=4; SE+9D, n=4; SE+7 W, n=3. Only the time points at which a sufficient number of CD68-positive cells were detected (>20 cells/mm2), allowing the percentage of Iba1^+^CD68^+^ cells to be meaningfully interpreted, i.e., from 1 day post-SE onward, were included in Figure **(M, N)**. Data are analyzed with Tukey’s test following One-way ANOVA (M) or with Dunn’s test following Kruskall-Wallis test (N). *: vs. SE+1D. Data are presented as the mean + SEM. *p<0.05; ***p<0.001; ****p<0.0001. Only significant differences between round and ramified cells are shown in **(K, L)**. All statistical tests are detailed in **Supplementary Table S2**.

We tracked the presence of infiltrated monocytes up to 7 weeks post-SE via CD68 and monitored their evolution into microglial-like cells on the basis of morphology and colabeling with the microglial marker Iba1 (**Figure 2**). CD68-positive round cells were predominantly found in the DG and CA1 regions, with a likely shift in peak entry between the two regions (**Figures 2K, L**). Only a few CD68-positive round cells were detected 7 weeks after SE, once epilepsy was established. Nine days after SE, CD68 colocalized with Iba1 in some non-round cells (**Figures 2D, I**), supporting the hypothesis that CD68-positive monocytes infiltrating the brain parenchyma after SE began to evolve into microglia-like cells. These morphologically microglia-like cells, both CD68-positive and Iba1-positive, were still detectable 7 weeks after SE (**Figures 2E, J, M, N**) during the chronic epilepsy phase, notably in CA1. In this manuscript, we refer to these cells as ‘monocytes’ upon entry, up to the 24-hour time point, and then as monocyte-macrophages (mo-mΦs) at later time points once their phenotype has evolved.

### 3.4 Infiltration of monocytes into other vulnerable regions subjected to massive neuroinflammatory processes after SE

Brain-infiltrating monocytes are observed not only in the hippocampus but also in the ventral limbic region (VLR), which includes (30) the amygdala, the piriform cortex, and the agranular insular cortex, as well as in the dorsal thalamus (ThD). These cells infiltrate these regions within 24 h post-SE, following the induction of the chemokine MCP1, and subsequently evolve into mo-mΦs, as described earlier for the hippocampus (**Supplementary Figure S6**). Notably, monocytes tend to accumulate in areas where the so-called ‘microglial scar’ is present, which is typically visualized via CD11b staining (**Supplementary Figures S6A–D**). These findings strongly suggest that the infiltrating monocytes remain in the brain parenchyma and transform into activated microglia-like monocyte-macrophages, which very likely constitute the cells that form the microglial scar (**Supplementary Figure S6**).

**Figure 3:**
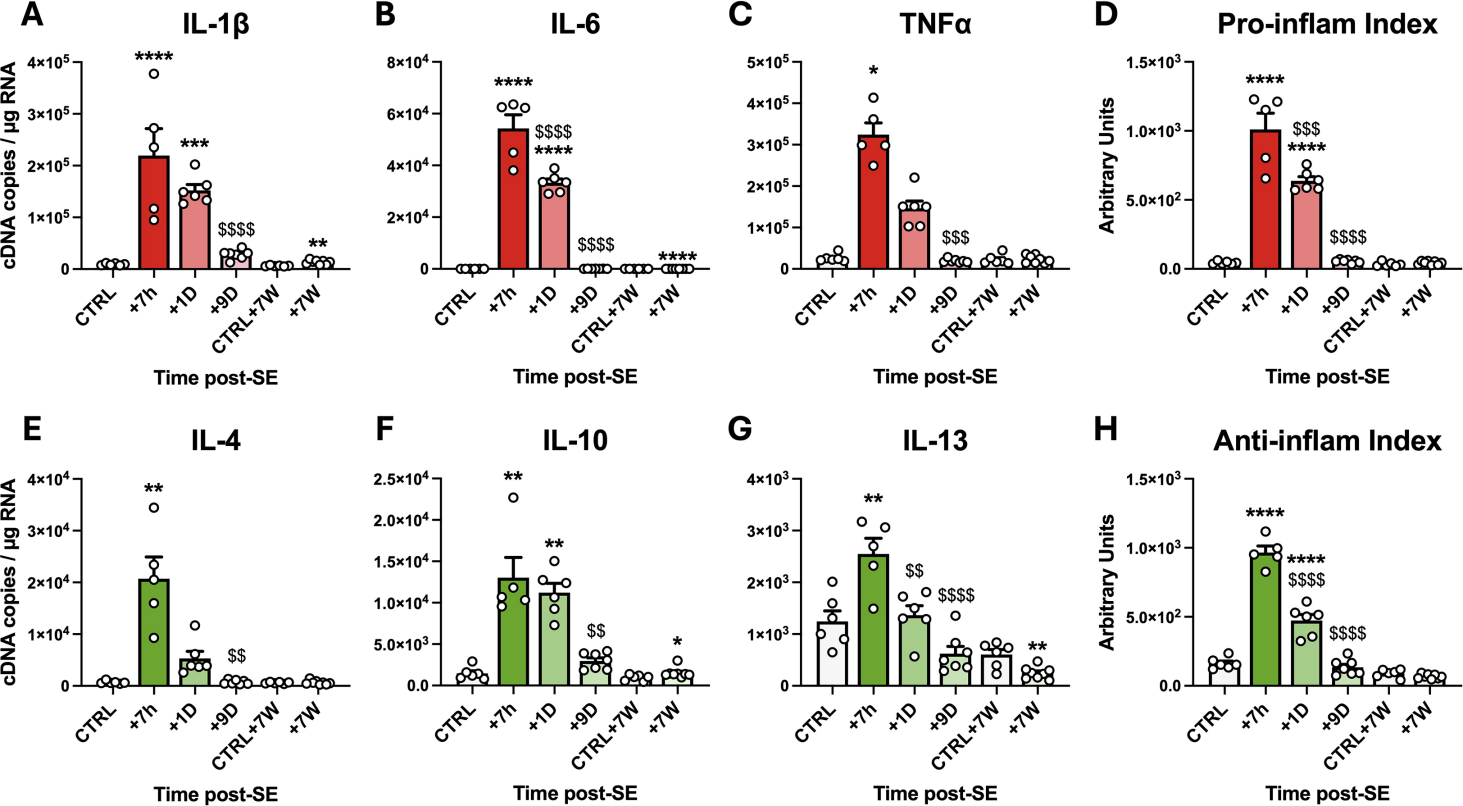
Transcriptional inflammatory response in the hippocampus, VLR and ThD during epileptogenesis and chronic epilepsy following SE induced by pilocarpine at P42 in rats. **(A–C)** Pro-inflammatory cytokine mRNA levels were quantified via calibrated RT‒qPCR in the hippocampi, ventral limbic region (VLR) and dorsal thalamus (ThD) following SE (CTRL, n=6; SE+7 h, n=5; SE+1D, n=6; SE+9D, n=7; CTRL+7 W, n=6; SE+7 W, n=8). **(D)** The proinflammatory index (PI-I) was calculated as described in the Methods section for IL-1β, IL-6 and TNFα. **(E–G)** Anti-inflammatory cytokine mRNA levels were also quantified in the same samples. **(H)** The anti-inflammatory index (AI-I) was calculated as described in the Methods section for IL-4, IL-10 and IL-13. The transcript levels of each gene were measured for each structure, averaged for each individual and expressed as cDNA copies per µg of total RNA. The levels by structure are detailed in **Supplementary Figure S7**. The PI-I and AI-I are expressed in arbitrary units. The data measured 7 h, 24 h and 9 days post SE were compared with those of P42 healthy controls, and those measured 7 weeks post SE were compared with those of another age-matched control group. Details of the statistical tests are presented in **Supplementery Table S2**. All the data are presented as the means ± SEMs. *: vs. the respective CTRL; $: vs. SE+7 h. 1 symbol, p<0.05; 2 symbols, p<0.01; 3 symbols, p<0.001; 4 symbols, p<0.0001.

To determine the respective contributions of microglia, monocytes/mo-mΦs and other nonmyeloid cells to the inflammatory response, we first characterized the tissue inflammatory status at the transcriptional level via RT–qPCR of homogenized brain tissue samples. We then investigated these inflammatory markers in FACS-sorted cell populations. To increase the cell yield and optimize the likelihood of obtaining a sufficient number of cells, particularly at the +7-week timepoint for subsequent RT–qPCR studies, sorting was performed on pooled samples from the three regions identified with the highest monocyte infiltration: the hippocampus, the VLR, and the ThD. To ensure comparability between tissue and sorted cell results, we present tissue-level quantifications as the average of the three regions (**Figure 3**), with detailed structure-specific data shown in **Supplementary Figure S7**.

The results revealed a sharp but transient increase in the transcript levels of the proinflammatory cytokines IL-1β, IL-6 and TNFα, which peaked 7 h after SE onset (**Figures 3A–C**; details of all the statistical tests are available in **Supplementary Table S2**). The transcript levels of these proinflammatory cytokines were still higher than those quantified in healthy controls 24 h after SE. IL-1β, IL-6 and TNFα transcripts returned to control levels 9 days after SE. However, the IL-1β and IL-6 transcript levels were still significantly elevated (222.8 ± 35.2% and 117.1 ± 2.1% of those in the respective controls) at 7 weeks post-SE during the chronic phase of epilepsy. The proinflammatory index (PI-I; see the Materials and Methods section) was calculated from these prototypic cytokine transcript levels to reflect the overall proinflammatory response after SE (**Figure 3D**). PI-I peaked at 7 h post-SE and was still significantly greater than that of the controls 24 h post-SE. The inflammatory response was also accompanied by marked and transient expression of anti-inflammatory cytokines, as evidenced by elevated levels of IL-4, IL-10 and IL-13 transcripts at 7 h and 24 h post-SE (**Figures 3E–G**). As for PI-I, an anti-inflammatory index (AI-I) was calculated from these prototypic cytokine transcript levels to summarize the general anti-inflammatory response after SE (**Figure 3H**). The AI-I peaked at 7 h and 24 h after SE and returned to control levels at 9 days post-SE.

### 3.5 Microglia are major contributors to pro-inflammatory cytokine expression after SE

**Figure 4:**
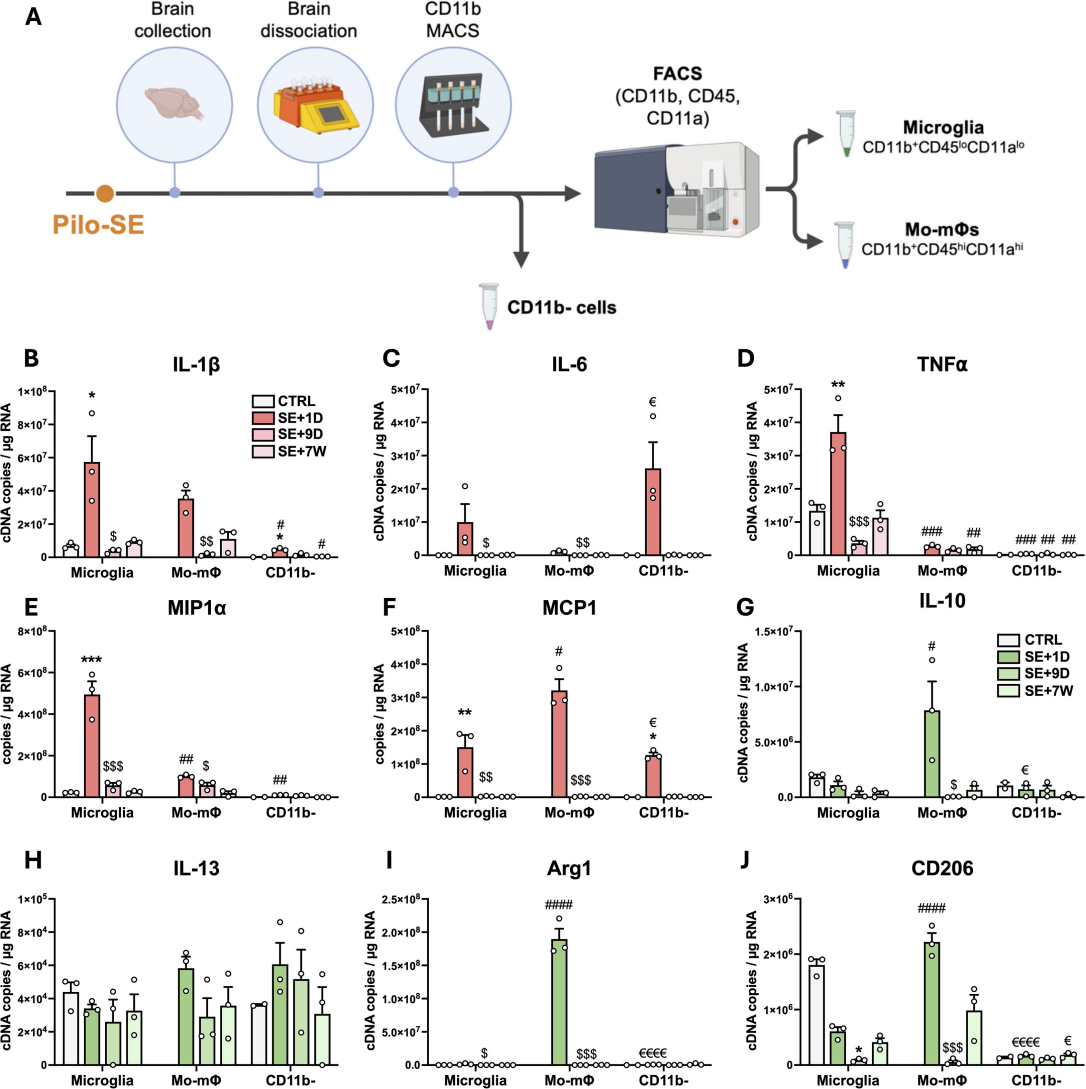
Transcriptional differences between sorted microglia, monocytes/mo-m@s and CDIIb-negative cells after SE. **(A)** CD11b- cells, microglia and monocytes/mo-mΦ were sorted via MACS and FACS. **(B-J)** Pro-inflammatory cytokine (IL-1ß, IL-6, and TNFα), chemokine (MIP 1α and MCP1) and immunomodulatory marker (IL-10, IL-13, Arg1 , and CD206) transcript levels were quantified by RT-qPCR in sorted cell populations (CTRL, n=2-3; SE+I D, n=3; SE+9D, n=3; CTRL 7 W, n=3; SE+7 W, n=3). Data measured 7 h, 24 h and 9 days post SE were compared to those of P42 healthy controls ("CTRL"), and those measured 7 weeks post SE were compared to those of another age-matched control group ("CTRL+7 W"). Details of the statistical tests are presented in **Supplementary Table S2**. The results are presented as the mean ± SEM. "Time post-SE" factor: * , vs. respective CTRL; $, vs. respective SE+1D; "cell type" factor: #, vs. respective microglia; €, vs. respective mo-mΦ. 1 symbol, p<0.05; 2 symbols, p<0.01; 3 symbols, p<0.001; 4 symbols, p<0.0001.

FACS was used to sort microglia (CD11b^+^CD45^lo^CD11a^lo^) and monocytes/mo-mΦs (CD11b^+^CD45^hi^CD11a^hi^) (31, 32), followed by CD11b enrichment via MACS. CD11b-negative cells (remaining brain cells, i.e., neurons, astrocytes, oligodendrocytes, endothelial cells, etc.) were also collected (**Figure 4A**). The hippocampus, VLR and ThD were microdissected and pooled for FACS studies as brain regions where monocyte infiltration was strong (**Supplementary Figure S6**). The study was performed at different times post-SE, i.e., 24 h, 9 days and 7 weeks post-SE. The entire tissue dissociation and cell sorting protocols of this study were performed in the presence of transcription and translation inhibitors in all buffers used to restrain *ex vivo* cell activation and obtain the most reliable inflammatory profile possible (24). In control rats, almost no CD11b^+^CD45^hi^CD11a^hi^ (monocytes/mo-mΦs) cells were detected (**Supplementary Figure S8**). The highest proportion of monocytes/mo-mΦs was quantified 24 h after SE. Furthermore, in line with what we observed at the histological level, we report the long-term presence of brain mo-mΦs in epileptic tissue, the proportion of which was still significantly greater than that measured in controls 7 weeks after SE (**Supplementary Figure S8F**).

We quantified the transcript levels of the myeloid cell markers CD11a and CD11b, whose corresponding proteins were used for cell sorting to distinguish microglia (CD11b^+^CD11a^lo^) from monocytes/mo-mΦs (CD11b^+^CD11a^hi^), via RT–qPCR of the sorted cell populations. Additionally, we measured the transcript levels of the microglial marker Iba1 and the monocyte marker CD68. Although we anticipated that CD11b mRNA would be expressed in both microglia and monocytes, we expected Iba1 mRNA to be restricted to microglia and that the CD11a and CD68 mRNAs would be exclusive to monocytes/mo-mΦs. However, this was not the case, as all four mRNAs were detected at nearly similar levels in both cell populations, with only occasional statistically significant differences (**Supplementary Figure S9**).

The transcript levels of 3 prototypical pro-inflammatory cytokines (IL-1β, IL-6 and TNFα), 2 chemokines (MIP1α and MCP1) and 4 immunomodulatory-associated genes (IL-10, IL-13, Arg1 and CD206) were quantified via RT–qPCR in the sorted cell populations from 24 h, i.e., once monocytes had infiltrated the brain, to 7 weeks post SE (**Figures 4B–J**).

IL-1β transcript levels were significantly greater in microglia sorted from brains collected 24 h after SE than in control microglia (**Figure 4B**). At this timepoint, IL-1β transcript levels were also detected at comparable levels in infiltrating monocytes and were slightly increased in CD11b-negative cells. IL-1β transcript levels returned to basal levels 9 days after SE. The highest levels of the IL-6 transcript were detected in CD11b-negative cells 24 h after SE (**Figure 4C**). These values were significantly greater than those measured in infiltrating monocytes but not in microglia. Compared with those in control microglia, TNF*α* transcript levels were increased in microglia sorted from brains collected 24 h after SE and were significantly greater than those measured in monocytes, with copy numbers almost 14-fold greater in microglia (**Figure 4D**). At 7 weeks post-SE, TNF*α* transcript levels were still 6-fold higher in microglia than in mo-mΦ. At each timepoint studied, the levels were greater in microglia than in CD11b-negative cells. The transcript level of the inflammatory chemokine MIP1*α* was significantly increased at 24 hours in microglia, reaching a level 5 times greater than that in mo-mΦ (**Figure 4E**). The mRNA level of MCP1, a chemokine primarily involved in monocyte recruitment, was strongly and transiently induced 24 h post-SE across all three sorted cell populations, with the highest level measured in monocytes (**Figure 4F**).

Overall, microglia appear to be the most pro-inflammatory cells during the intense inflammatory response observed during epileptogenesis, returning to a baseline level during the chronic phase of epilepsy. Monocytes make a smaller contribution to the expression of pro-inflammatory cytokines during epileptogenesis, primarily by expressing IL-1β 24 h after SE induction. The Mo-mΦs that remained in the tissue at 7 weeks presented low levels of IL-1β transcript expression. Consequently, the residual low-grade tissue inflammation observed at 7 weeks post-SE might be sustained by mo-mΦs that have become integrated into the brain parenchyma over the long term.

### 3.6 Brain-infiltrating monocytes exhibit an anti-inflammatory phenotype upon entry

The significant induction of anti-inflammatory cytokines at the tissue level at 7 hours post-SE (**Figures 3E–G**) cannot be attributed to infiltrating monocytes, as these cells only appear in brain tissue after this timepoint. By 1 day post-SE and beyond, their presence in brain tissue prompted an essential investigation into how the anti-inflammatory response is partitioned among microglia, mo-mΦs, and other brain cells. IL-10 transcript levels remained stable in microglia and in CD11b-negative cells following SE but were high in infiltrating monocytes 24 h after SE, suggesting that infiltrating monocytes are the main contributors to the increase in IL-10 transcript levels observed at the tissue level 24 h after SE (**Figure 4G**). IL-13 transcript levels also remained stable in microglia and in CD11b-negative cells following SE and were detected in monocytes/mo-mΦs at similar levels (**Figure 4H**). The transcript level of Arg1, a characteristic marker of the “M2” anti-inflammatory phenotype, was dramatically greater in infiltrating monocytes 24 h after SE than in microglia and CD11b-negative cells (**Figure 4I**). The transcript levels of the immunomodulator marker CD206 decreased following SE in microglia. At 24 h post-SE, the transcript level of CD206 was ∼ 4-fold greater in infiltrating monocytes than in microglia (**Figure 4J**). Taken together, these results suggest that monocytes play a significant role in the establishment of the anti-inflammatory response upon their entry 24 h post-SE and transiently adopt a neuroprotective phenotype.

## 4 Discussion

The role of infiltrating monocytes in the robust inflammatory response following epileptogenic insults, such as SE, in comparison with that of resident microglia, as well as their long-term fate within brain tissue, remains a subject of ongoing debate.

In P42 rats, pilocarpine-induced SE triggered a strong inflammatory reaction not only in the hippocampus but also more broadly in the ventral limbic and thalamic regions of the brain within hours. At the cellular level, the inflammatory response is characterized by the early transient activation of microglia. Peripheral monocytes infiltrated the hippocampus in significant numbers between 7 h and 9 days after the onset of SE. Their number peaks at 24 h, but some remain detectable for several weeks post-SE. Monocyte engraftment and their evolution into brain mo-mΦs was evidenced histologically over the long term, with a phenotype resembling that of microglia. We demonstrated that mo-mΦs play a significant role in the formation of the glial scar in hippocampal subregions, particularly the CA1 region and the DG. This scar is a well-known characteristic of hippocampal sclerosis that is commonly observed in various experimental models and patients with TLE and has long been believed to be primarily composed of resident microglia (33, 34). It could play an adaptive role by causing inflammatory damage and preserving the integrity of the surrounding tissue. The prolonged persistence of microglial activation in the CA1 region, compared with that in the DG, could suggest region-specific differences in the ability of microglia to regulate tissue damage in relation to the resolution of inflammation. It is also possible that this different level of CD11b activation may be a signature of a distinction between resident microglia and myeloid cells of peripheral origin, which may have established themselves more durably in certain regions rather than others.

At the molecular level, while neuroinflammation largely resolves within a few days, low-grade, persistent inflammation is observed during the chronic phase of epilepsy. Massive activation of both pro- and anti-inflammatory genes was detected at the mRNA level. IL-1β and IL-6 transcript levels were still significantly elevated up to 7 weeks post-SE during the chronic phase of epilepsy. Data collected via FACS revealed that microglia were the primary contributors to the early inflammatory peak, whereas monocytes exhibited a more prominent M2-type, anti-inflammatory and neuroprotective state than did microglia at 24 h after SE. However, once they evolve into mo-mΦs, they might be the main contributors to the persistent low-grade inflammation observed during chronic epilepsy.

Our findings contrast with previous reports by Vinet et al. (11) and Varvel et al. (10), which described infiltrating myeloid cells as highly pro-inflammatory during epileptogenesis (10, 11). In the pilocarpine-induced SE model, Vinet and colleagues observed that hippocampal microglia remained relatively immunosuppressed, whereas infiltrating myeloid cells displayed a strong inflammatory profile in the early phase of epileptogenesis. Similarly, in the kainic acid-induced SE model, Varvel et al. reported comparable IL-1β levels in FACS-isolated microglia and monocyte-derived macrophages (mo-mΦs) following SE, but higher TNFα expression in mo-mΦs than in activated microglia. Notably, neither of these studies examined anti-inflammatory markers in mo-mΦs. In contrast, in our study, we found that, at the peak of infiltration, monocytes transiently exhibit an overall anti-inflammatory and neuroprotective phenotype, challenging the prevailing view that infiltrating monocytes are primarily inflammatory during this stage. Measuring the inflammatory profile of these cells is inherently difficult because distinguishing them from resident microglia typically requires a cell sorting step, and standard tissue dissociation protocols are known to induce substantial transcriptional and translational changes *ex vivo* (24, 36). In our study, we implemented an optimized brain dissociation and cell-sorting protocol designed to minimize *ex vivo* cell activation and simultaneously achieve sufficient cell yields to obtain reliable and interpretable data. To minimize such *ex vivo* artifacts, all samples had to be processed simultaneously in the presence of transcription and translation inhibitors, an approach that necessarily constrained the achievable sample size in these experiments. Despite this limitation, statistical power remained sufficient to support our conclusions. In the chronic phase of epilepsy, when microglia have largely returned to a baseline state in terms of cytokine expression and morphology, a subset of mo-mΦs persists in the parenchyma and continues to express low levels of inflammatory cytokines. We therefore hypothesize that these monocytes, because of their presence and not because they express higher levels of pro-inflammatory markers than microglia do, fuel the persistent low-grade inflammation observed once epilepsy is established.

To establish the peripheral origin of the round cells observed 24 hours after SE, confirming the entry of monocytes into the brain tissue, we employed a fluorescence tracing technique through intravenous administration of FYG particles (29). This approach requires the depletion of peripheral myeloid cells before SE onset, which means that at the time of SE, the monocyte pool is not completely replenished, potentially leading to a reduction in the number of infiltrating cells compared with the normal condition. This diminished number of infiltrating monocytes may account for the aggravated phenotype we observed in comparison to that in rats subjected to standard SE induction. This finding is in line with the observation that preventing monocyte infiltration resulted in dampened hippocampal neurodegeneration in a kainate-SE mouse model (12) and was detrimental in models of stroke (37, 38) and Alzheimer’s disease (39).

In our study, the extended observation of monocytes and subsequent mo-mΦs relied on tracking of cellular markers, notably CD68 and CD11a. However, it is still possible that some monocyte subpopulations do not express these markers. Additionally, our data do not provide definitive insights into whether the decline in mo-mΦ numbers over time is attributable to alterations in the expression of the measured markers or actual elimination of cells. Once a sufficiently specific marker of mo-mΦs is fully validated, the use of genetic models will allow us to address these questions reliably by conducting long-term fate mapping studies. Notably, while no CD68 or CD11a protein labeling was detected in the brain under control conditions, their transcripts were detectable in microglia, making them unsuitable as reporter genes for the establishment of a transgenic model. However, Arg1 was found to be entirely monocyte-specific but was transiently expressed only upon its entry into brain tissue. Arg1 has also been shown to be specifically expressed by infiltrating myeloid cells in the central nervous system in models of spinal cord injury and multiple sclerosis (19). The Arg1 promoter could therefore be considered for inducible expression of a reporter gene up to 24 h after SE. Future single-cell screening analysis will allow for the identification of other monocyte-specific genes.

Investigation of differences in expression profiles between microglia and monocytes/mo-mΦs was not carried out at the single-cell level but rather in whole populations separated by FACS after CD11b-MACS enrichment. Thus, our results do not allow us to ascertain whether all cells within a given population share the same inflammatory state or if they comprise multiple subpopulations exhibiting varying levels of activation, with the latter hypothesis being the more likely scenario, considering the heterogeneity of both microglia and monocytes (40–42).

**Figure 5:**
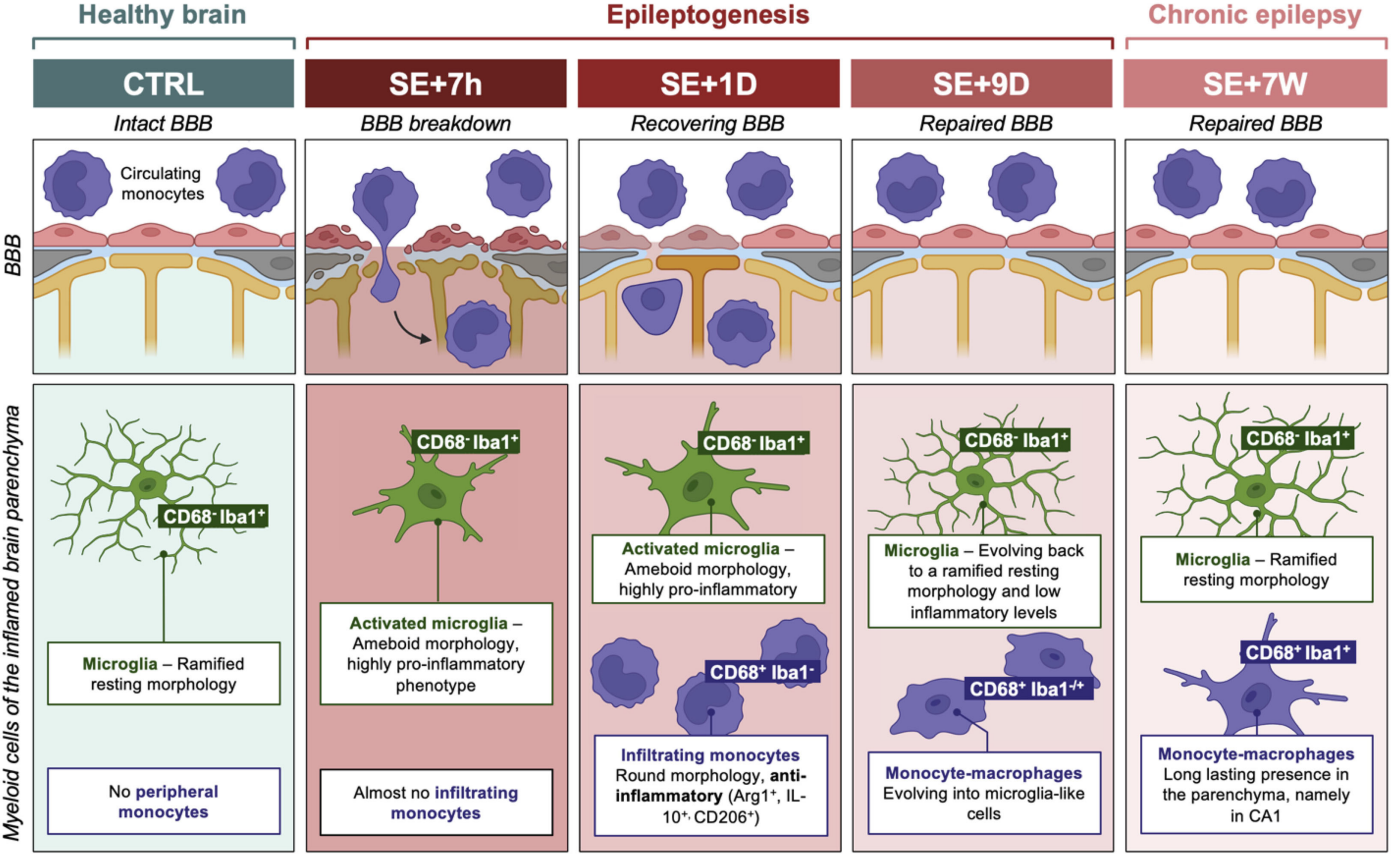
Proposed time course of entry and integration of monocytes infiltrating inflamed brain regions after pilocarpine-induced SE in rats. The blood—brain barrier (BBB) state following SE is depicted in the boxes at the top of the diagram (35). Myeloid cells in the hippocampus, the VLR and the ThD parenchyma, i.e., microglia and monocytes, are represented in the boxes at the bottom. Markers enabling the staining of both cell types at the histological level (but not at the transcript level) are highlighted in green for microglia and purple for monocytes/monocyte-macrophages (mo-mΦs). At 7 hours after SE, microglia are primarily responsible for the extensive expression of proinflammatory cytokines, and monocytes have not yet invaded brain tissue. By 24 hours after SE, while inflammation remains at a significant level, a substantial number of round monocytes are observed, contributing to the expression of IL-1ß but displaying a predominantly neuroprotective phenotype. Nine days after SE, a glial scar composed mainly of microglia and brain mo-mΦs formed, although the inflammatory response was largely resolved. At this stage, some mo-mΦs start to coexpress the monocyte marker CD68 and the microglia marker Iba1. During the chronic epilepsy phase, microglia return to a basal inflammatory state, but low-grade inflammation persists, likely due to the presence of mo-mΦs. BBB, blood—brain barrier; CTRL, control, resting condition; D, day; SE, status epilepticus; W, week.

Conclusions – Taken together, our findings allow us to propose a time course depicting the contribution of microglia and infiltrating monocytes/mo-mΦs to inflammation following pilocarpine-induced SE in rats, from early epileptogenesis to chronic epilepsy (**Figure 5**). Importantly, our results challenge the traditionally held view of mo-mΦs as major proinflammatory contributors in this context. By carefully limiting *ex vivo* cellular activation, we revealed that microglia, rather than mo-mΦs, are the primary drivers of the pro-inflammatory response. This repositioning of roles not only enhances our understanding of post-SE inflammation but also underscores the need to refine therapeutic strategies targeting these distinct cellular populations. Specifically, infiltrating monocytes emerge as promising targets, either by enhancing their anti-inflammatory phenotype during the early phase of epileptogenesis or by reactivating it once epilepsy is established.

## Data Availability

The original contributions presented in the study are included in the article/**Supplementary Material**. Further inquiries can be directed to the corresponding authors.
